# Models for Predicting the Biomass of *Cunninghamialanceolata* Trees and Stands in Southeastern China

**DOI:** 10.1371/journal.pone.0169747

**Published:** 2017-01-17

**Authors:** Mei Guangyi, Sun Yujun, Sajjad Saeed

**Affiliations:** Laboratory for Silviculture and Conservation, Beijing Forestry University, Beijing, China; Chinese Academy of Forestry, CHINA

## Abstract

Using existing equations to estimate the biomass of a single tree or a forest stand still involves large uncertainties. In this study, we developed individual-tree biomass models for Chinese Fir (*Cunninghamia lanceolata*.) stands in Fujian Province, southeast China, by using 74 previously established models that have been most commonly used to estimate tree biomass. We selected the best fit models and modified them. The results showed that the published model *ln*(*B*(*Biomass*)) = *a* + *b* * *ln*(*D*) + *c* * (*ln*(*H*))^2^ + *d* * (*ln*(*H*))^3^ + *e* * *ln*(*WD*) had the best fit for estimating the tree biomass of Chinese Fir stands. Furthermore, we observed that variables D(diameter at breast height), H (height), and WD(wood density)were significantly correlated with the total tree biomass estimation model. As a result, a natural logarithm structure gave the best estimates for the tree biomass structure. Finally, when a multi-step improvement on tree biomass model was performed, the tree biomass model with Tree volume(TV), WD and biomass wood density conversion factor (BECF),achieved the highest simulation accuracy, expressed as *ln*(*TB*) = −0.0703 + 0.9780 * *ln*(*TV*) + 0.0213 * *ln*(*WD*) + 1.0166 * *ln*(*BECF*). Therefore, when TV, WD and BECF were combined with tree biomass volume coefficient bi for Chinese Fir, the stand biomass (SB)model included both volume(SV) and coefficient bi variables of the stand as follows: *bi* = *Exp*(−0.0703+0.9780*ln(*TV*)+0.0213 * *ln*(*WD*)+1.0166*ln(*BECF*)). The stand biomass model is *SB* = *SV*/*TV* * *bi*.

## Introduction

Forest managers are constantly facing new problems and challenges, which include climate change, mitigation and adaptation[1]. Accurate and precise measurements of forest ecosystem parameters such as biomass will be important for future forest management[2–3]. In addition to climate change, the development of a regional biomass energy industry and artificial forests means that the energy management problems will still exist, so highly accurate forest stand biomass models is of key importance[4].

Current biomass equations mainly use the following methods: the biomass factor method, the allometry growth equation method and the volume source biomass method[5].At present, many forest biomass estimation models primarily use the diameter at breast height (D) to estimate the biomass[6]. However, this method lacks specificity for different tree species and site features and the accuracy of the area measurement is always poor, resulting in high precision on only a small scale.

Using different allometric growth equation methods, Jennifer et al. have incorporated data from published studies into new biomass estimation equations[6].To adapt them to different research purposes, many researchers have recently performed many trials and modified various models[5]. In previous studies, Li et al. and Dimitris et al. summarized biomass models that use the diameter at breast height (D), tree height (H), D^2^H and DH as the independent variables[7–8]. They used a combination of the commonly used power function model, an exponential model and a polynomial model to simulate a portion of or the whole plant wood biomass. Similarly, Liu et al. conducted a relevant analysis of the biomass of shrub using a new biomass model[9]. Almeida et al. included the related parameter D^2^in a biomass analysis [10]. As biomass research and utilization progressed, José established the site index (SI) and forest biomass variable model of the stand basal area[11]. This study showed that as the objective changed, the reliability of the D indicator did not meet the needs of practical forestry estimates. Wood density (WD) and stand basal area (G)have become increasingly popular. For example, Daniel et al. and Sabina et al. used a combination of D, H and WD to establish a logarithmic and exponential biomass model that used a combination of these indicators[12–13].Timothy et al. used a fusion variable and a logarithmic model to estimate the biomass of the Amazon forest[14].To study the structural relationships between form factor, wood density, and biomass in African savanna woodlands, Matthew et al. established a variable containing D, H, WD and G in a logarithmic combined biomass model[15].

Several studies have asserted that, at a small scale, a greater number of independent variables can increase the accuracy of the model’s estimation of biomass[14–16].Thus, large-scale forest biomass estimates consider the use of binary and tertiary biomass models, which is necessary to obtain a more accurate estimate [16]. Therefore, in the context of different purposes and the actual demand, an increase in the magnitude of an independent variable of the biomass model is important [17]. In many cases, however, when a model was used to assess biomass, the evaluation accuracy for large or small areas was not high, or uncertainty or restrictions were present [6]. For instance, the definition of a forest stand is uncertain at a large or a small scale. Thus, the use of either scale leads to uncertainty when a model is selected [18]. To solve this problem, Zuo et al. used different parameters to analyze a model to estimate the biomass of Fir forests[19]. Esteban et al. used D and H as independent variables to determine 8 parameters in a forest stand biomass model[20].

Chinese Fir is one of the most popular plantation timber species in China because it has good quality timber, grows rapidly, has a straight stem and is highly resistant to bending[21–22].To evaluate the stand biomass of a Chinese Fir forest on a large scale, a model must be extended to the entire stand or planted region for an accurate estimate of the biomass [23].Because an established forest biomass model may not be suitable for a Chinese Fir stand, a more appropriate stand variable also needs to be determined [20]. Studies of Chinese Fir stand biomass showed that a model based on a large sample of forest biomass had a relatively high accuracy and could be applied to a large area, but a regional model that considered a small sample was limited to a restricted area.

The specific objectives of this study were (1) to select and modify the single tree biomass model with highest accuracy for Chinese Fir via a comprehensive comparison and analysis of current biomass models and (2) to calculate a more appropriate conversion coefficient for the estimation of Chinese Fir biomass on a stand scale.

## Materials and Methods

### 2.1 Materials

The study area was in the Jiangle state-owned forest farm located between 117°05′-117°40′E and 26°26′-27° 04′ N, Fujian province, China. This forest farm has a designated study area, and these forest lands are all experimental plantations. The Jiangle state-owned forest farm produces Chinese Fir wood. The forest farm covers a large area and experiences high levels of wood trading. No permissions were required to study in this area, which is one reason that we selected it. The primary species in the forest farm include Chinese Fir, Masson pine, and Moso bamboo. Many studies have been published using data collected from the Jiangle forest farm.

**Fig 1:**
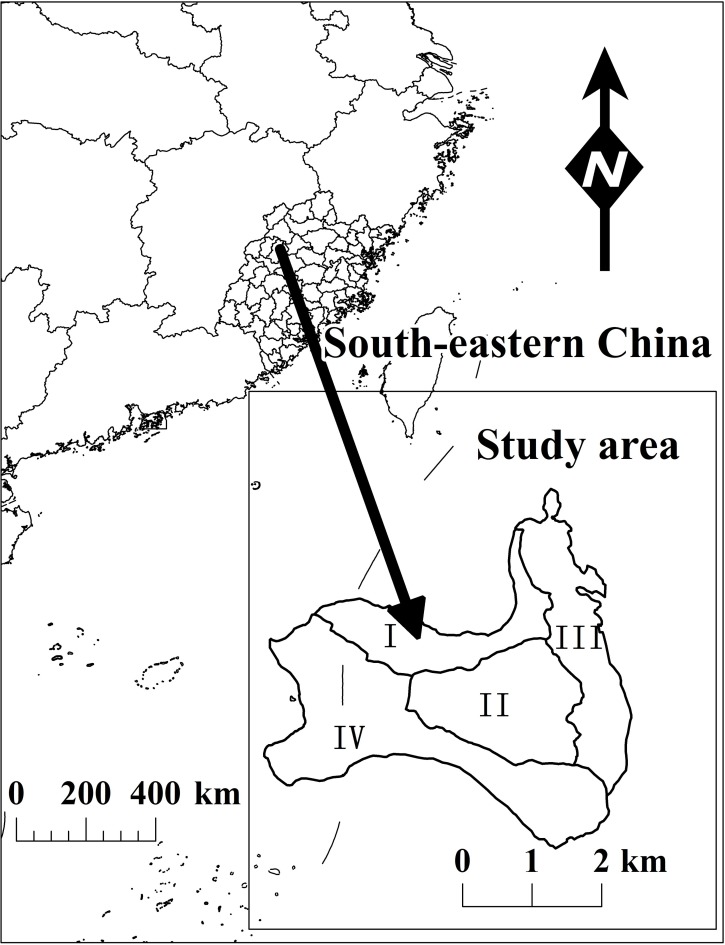
Four sites in Fujian province, Southeast China, where 35 trees were sampled.

The region is characterized by red soil and has a mean annual precipitation of approximately 1699 mm, a mean annual frost-free season of 287 days, and a mean annual temperature of 18.7°C. We sampled four regions, which were divided into 35 plots of Chinese Fir trees and were designated as I, II, III and IV (Fig 1). The plots were established between 2010 and 2014 and vary in size from 400 to 600 m^2^.

We measured the diameter at breast height (DBH) over the bark (at 1.3 m above ground) of fresh trees (height > 1.3 m) and the total tree height of 35 trees that were felled for stem analysis. Before felling each tree, we measured two parameters: the diameter at breast height (1.3 m above ground) and the total tree height (H). After felling, we measured the diameter at intervals of 1 m above the breast height depending on the total tree height along the largest axis and smallest axis using a diameter tape. The base diameter of all sections was measured at intervals of 1 m. (1)The fresh mass of the stem wood, stem bark, branches, and foliage were measured, and subsamples were taken and weighed in the field. (2) The fresh mass of the stem bark was equal to the fresh mass of the stem or the trunk multiplied by the bark percent in the subsamples. (3)The whole roots were excavated, and the fresh weight of the stump (below ground level), the coarse roots (greater than 10 mm), the middle roots(2–10 mm) and the small roots (0–2 mm) were measured, and subsamples were taken [3].The subsamples were used for the determination of the fresh to dry weight ratio (65°C). Based on the ratio of the dry biomass to the fresh biomass, the biomass of the stem, bark, foliage and roots were calculated and summed to obtain the total biomass of each tree (TB). Table 1 summarizes the characteristics of the selected trees.

**Table 1:** Mean diameter at breast height (1.3)(D), total height (H), age, BECF (*BCEF* = *BEF* * *WD*), where BEF is the biomass expansion factor), volume(V), wood density (WD), total tree biomass (TB) for sampled biomass trees.

	D(cm)	H(m)	Age	BECF	V (m^3^)	WD	B (kg)
**Mean**	17.0	15.8	24.4	391.8	0.2655	304.2	107.8
**SD**	7.3	6.7	9.5	81.4	0.31	59.7	101.3
**Minimum**	5.1	4.1	6	236.3	0.0060	117.0	4.6
**Maximum**	38.4	31.8	38	613.8	1.7091	427.1	482.4

### 2.2 Model fitting and evaluation

A total of 74 high-precision biomass models were selected from a large number of previously published biomass estimation models[8,24–25]. The non-linear least squares regression (nls) function was used to fit the equations using R project. Different starting values were used for the parameters to ensure that a global minimum was achieved.

The best function was selected on the basis of the following four statistical criteria: the mean absolute bias (MAB), the root mean square error (RMSE), the average relative error (ARE) and the adjusted coefficient of determination (R^2^) [26–27]. The formulae for these statistics are as follows:

$$MAB=\frac{\sum_{i=1}^{n}\left|\left(B_{i}-{\hat{B}}_{i}\right)\right|}{n}$$ (1)

$$RMSE=\sqrt{\frac{\sum_{i=1}^{n}{\left(B_{i}-{\hat{B}}_{i}\right)}^{2}}{n-1}}$$ (2)

$$ARE=\sum (\left|(B_{i}-\hat{B})/B_{i}\right|)/n*100\%$$ (3)

$$R^{2}=1-\frac{\sum_{i=1}^{n}{\left(B_{i}-{\hat{B}}_{i}\right)}^{2}}{\sum_{i=1}^{n}{\left(B_{i}-\bar{B}\right)}^{2}}$$ (4)

where *B*_*i*_ and ${\hat{B}}_{i}$ are the biomass measurements and predictions, respectively, $\bar{B}$ is the average of the measurements, and n is the number of data points.

### 2.3 Variables computed

1. V (volume):Based on a taper model, formula (5) was used to calculate the volume of the trees [28], as follows:
  $$V=\frac{\prod }{40000}\int_{0}^{H}D^{2}{\left(\frac{\left(H-h\right)}{\left(H-1.3\right)}\right)}^{\left(3.482321-2.153699*h^{0.007}\right)}dh.$$ (5)
2. BEF (biomass expansion factor) [29]:
  BEF=Abovegroundbiomass/Trunkbiomass. (6)
3. WD(wood density):
  $$WD=Aboveground\;biomass/Stem\;dry\;weight\;\left(kg*m^{-3}\right).$$ (7)
4. BECF(biomass wood density conversion factor) [30]:
  BCEF=BEF*WD. (8)
  where V is the tree volume, H is the total height, D is the diameter at breast height, and h is the height above ground level. BCEF is the biomass wood density conversion factor, that is, the ratio of the aboveground biomass to the stem volume (kg*m^-3^). BEF is the biomass expansion factor, that is, the ratio of aboveground biomass to the trunk biomass, and is dimensionless. WD is the wood density, that is, the dry weight per unit volume of wood (kg*m^-3^).

We used a jackknife model validation method.

## Results

### 3.1 Selection of a total tree biomass model

**Fig 2:**
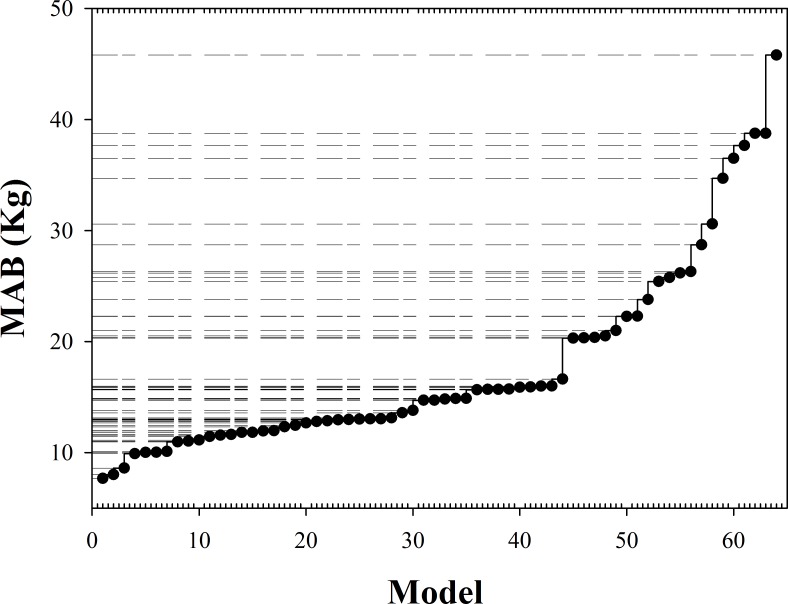
The MAB of 64 convergence biomass models in Table 2.

The best method to calculate the total tree biomass (both aboveground and belowground)can be observed from the results in Table 2. Based on a model accuracy evaluation variable analysis, the MAB in model No.1 was the lowest of the candidate models (Fig 2). The ARE of model No.1 was 7.037, 12.623 for model No.2, and 15.931 for model No.3, indicating that model No.1 produced the best simulation (Table 2).

**Table 2:** Seventy-fourpreviously published and commonly used biomass models.

No	Model	a	b	c	d	e	MAB	RMSE	R^2^
1	*ln*(*B*) ∼ *a* + *b* * *ln*(*D*) + *c* * (*ln*(*H*))^2^ + *d* * (*ln*(*H*))^3^ + *e* * *ln*(*WD*)	-5.744	2.480	-0.217	-0.278	0.60	7.675	13.656	0.982
2	*B* ∼ *exp*(*a*) * (*D* + 1^*b*^ * *H*^*c*^ * *exp*(*d* * *D*) * *exp*(*e* * *H*)	-6.104	5.162	-1.340	-0.138	0.10	8.017	11.750	0.987
3	*B* ∼ *exp*(*a*) * (*D* + 1)^*b*^ * *H*^*c*^ * *exp*(*d* * *D*)	-6.250	3.389	0.704	-0.064		8.601	12.602	0.985
4	*B* ∼ *a* + *b* * *D* + *c* * *D*^2^ + *d* * *H* + *e* * *D* * *H*	-2.878	4.827	-0.124	-7.493	0.60	9.892	13.796	0.981
5	*ln*(*B*) ∼ *a* + b*ln(*D*^2^ * *H*) + c*ln(*WD*)	-4.720	0.831	0.370			10.007	18.936	0.967
6	*ln*(*B*) ∼ *a* + b*ln(*D*) +c*ln(*WD*)	-5.702	2.546	0.504			10.025	19.425	0.965
7	*ln*(*B*) ∼ *a* + b*ln(*D*) +c*ln(*H*) + d*ln(*WD*)	-5.723	2.567	-0.020	0.507		10.095	19.895	0.965
8	*B* ∼ *a* + *b* * *D* + *c* * *D*^2^ + *d* * (*D*^3^ / *H*)	-9.452	-0.808	0.572	-0.171		10.961	14.627	0.979
9	*B* ∼ *a* + *b* * *D*^2^ + *c* * *D* + *d* * *D* * *H*	-10.924	-0.696	0.198	0.200		11.029	14.847	0.979
10	*B* ∼ *a* + *D*^2^ * *b* + *D* * *H* * *c*	-16.477	0.195	0.183			11.130	14.894	0.978
11	*ln*(*B*) ∼ *a* + (*D* / (*D* + 10)) * *b*	-2.411	10.864				11.440	15.666	0.976
12	*ln*(*B*) ∼ *a*+b*(*D*/(*D*+7)+c*H+d*ln(*H*))	-2.785	10.899	0.005	-0.046		11.558	21.683	0.954
13	*ln*(*B*) ∼ *a*+(*D*/(*D*+11))*b+c*ln(*H*)	-2.121	10.451	0.071			11.628	16.219	0.974
14	*B* ∼ *exp*(*a*+b*ln(*D*^2^ * *H*))	-1.823	0.748				11.809	16.116	0.975
15	*B* ∼ a*(*D*^2^ * *H*)^*b*^	0.162	0.748				11.809	16.116	0.975
16	*B* ∼ a*D^*b*^ * *H*^*c*^	0.171	1.574	0.650			11.943	15.834	0.976
17	*ln*(*B*) ∼ *a*+b*(*D*/(*D*+11))	-2.111	10.757				11.970	16.732	0.973
18	*B* ∼ *exp*(*a*) * (*D*+1)^*b*^ * *H*^*c*^	-2.050	1.617	0.674			12.316	16.148	0.975
19	*B* ∼ *exp*(*a*+*b* * *ln*(*D*^2^ * *H* * *G*))	-1.543	0.436				12.455	16.285	0.975
20	*B* ∼ *a* + *b* * *H* + *c* * *D*^2^	-24.870	1.493	0.319			12.669	16.118	0.975
21	*ln*(*B*) ∼ *a*+b*D/(*D*+13)+c*H+d*ln(*H*)	-1.582	10.205	0.005	0.040		12.796	21.425	0.955
22	*B* ∼ *a* + *b* * *D*^2^	-11.692	0.349				12.853	16.582	0.973
23	*B* ∼ *a* + *b* * *D*^2^ * *H* + *c* * *D*^2^	-13.130	0.001	0.363			12.940	16.810	0.973
24	*B* ∼ *a* + *b* * *D* + *c* * *D*^2^ * *H*	-48.700	6.542	0.006			12.968	16.736	0.974
25	*B* ∼ *a* + *b* * *D*^*c*^	-20.336	0.559	1.869			13.007	16.442	0.974
26	*ln*(*B*) ∼ *a*+(*D*/(*D*+14))*b+c*ln(*H*)	-1.499	10.211	0.106			13.028	21.937	0.953
27	*ln*(*B*) ∼ *a*+(*D*/(*D*+13))*b	-1.643	10.667				13.037	20.706	0.958
28	*B* ∼ *a* + *b* * *D* + *c* * *D*^2^	-23.013	1.314	0.317			13.118	16.628	0.974
29	*ln*(*B*) ∼ *a*+(*D*/(*D*+14))*b	-1.456	10.666				13.578	23.191	0.948
30	*ln*(*B*) ∼ *a*+b*D/(*D*+18)+c*H+d*ln(*H*)	-1.338	10.419	-0.020	0.360		13.790	23.329	0.947
31	*B* ∼ *a* * *D*^*b*^	0.245	2.090				14.713	18.004	0.969
32	*B* ∼ *exp*(*a* + *b* * *ln*(*D*)	-1.407	2.090				14.713	18.004	0.969
33	*ln*(*B*) ∼ *a*+b*ln(*D*)+c*ln(H*D^2^)	-2.821	2.117	0.143			14.812	29.533	0.915
34	*ln*(*B*) ∼ *a*+(*D*/(*D*+5))*b	-5.560	13.001				14.864	24.769	0.940
35	*ln*(*B*) ∼ *a*+*b* * *ln*(*D*) + *c* * *H*+*d* * *ln*(*H* * *D*^2^)	-2.794	2.139	0.001	0.130		14.879	30.159	0.911
36	*ln*(*B*) ∼ *a*+*b* * *ln*(*D*) + *c* * *H*	-2.676	2.441	0.008			15.654	34.441	0.884
37	*ln*(*B*) ∼ *a*+*b* * *ln*(*D*)	-2.843	2.550				15.682	32.304	0.901
38	*ln*(*B*) ∼ *a*+*b* * *ln*(*pi* * *D*)	-5.762	2.550				15.682	31.826	0.901
39	*ln*(*B*) ∼ *a*+b*(*D*/(*D*+30)+c*H + d*ln(*H*))	-1.261	11.587	-0.005	0.074		15.714	28.420	0.921
40	*ln*(*B*) ∼ *a* + (*D*/(*D*+18))*b	-0.901	10.841				15.877	34.020	0.887
41	*B* ∼ a*(*WD* * *D*^2^ * *H*)/1000	0.054					15.900	35.951	0.878
42	*B* ∼ *a* + *b* * *H* + *c* * *D*^2^ * *H*	-24.710	4.595	0.008			15.983	20.399	0.961
43	*B* ∼ *a* + *b* * *D*^2^ * *H* + *c* * *H*^2^	1.586	0.007	0.197			15.996	20.989	0.958
44	*B* ∼ *a* + *b* * *D* + *c* * (*D*^2^ *H)^2^	-85.590	10.830	0.000			16.618	21.062	0.958
45	*B* ∼ *a* + *b* * *H*^2^ + *c* * *H*^3^	0.373	0.155	0.010			20.297	27.868	0.924
46	*B* ∼ *a* * *H*^*b*^	0.061	2.595				20.318	28.225	0.925
47	*B* ∼ *a* + *b* * *D* + *c* * *H*^2^	-81.275	7.637	0.200			20.367	26.335	0.934
48	*B* ∼ *a* * *V* + *b*	312.470	24.740				20.502	26.497	0.934
49	*B* ∼ *a* + *b* * *D*^2^ * *H*	27.464	0.011				20.986	27.187	0.930
50	*B* ∼ *a* + *b* * *D*	-118.191	13.264				22.249	29.696	0.917
51	*B* ∼ *a* + *b* * *D* + *c* * *H*	-115.504	15.366	-2.428			22.285	29.212	0.919
52	*B* ∼ *a* * *H* * *D*^2^	0.013					23.777	34.283	0.886
53	*B* ∼ *a* + *b* * *H* + c*(*D*^2^ * *H*)^2^	-64.280	9.985	0.000			25.411	30.737	0.911
54	*B* ∼ *a* + *b* * (1/*D*^2^ * *H*) * *D*^2^ * *H*	-23.380	0.445				25.779	31.257	0.905
55	*ln*(*B*) ∼ *a* + *b* * *ln*(*D*)^2^	0.405	0.484				26.178	81.410	0.355
56	*B* ∼ *a* * *exp*(*H* * *b*)	14.665	0.112				26.306	32.099	0.900
57	*B* ∼ *a* * *exp*(*b* * *D*)	23.845	0.081				28.721	32.615	0.899
58	*B* ∼ *a* * *BA*^*b*^ * *SI*^*c*^	1.067	0.604	1.206			30.605	61.748	0.640
59	*ln*(*B*) ∼ *ln*(*a*) + *b* * *H*	4.070	0.172				34.697	90.892	0.219
60	*B* ∼ *a* + *b* * *H*	-105.634	13.467				36.505	47.096	0.790
61	*B* ∼ *a* + *b* * *ln*(*D*)	-387.080	180.950				37.662	53.784	0.727
62	*B* ∼ *a* * *ln*(*H* * *D*^2^) + *b*	58.273	-365.449				38.751	55.563	0.699
63	*B* ∼ *a* + *b* * *ln*(*D*^2^ * *H*)	-365.451	58.274				38.751	56.399	0.699
64	*B* ∼ *a* + *b* * *ln*(*H*)	-295.080	151.940				45.808	64.782	0.603
65	*ln*(*B*) ∼ *ln*(*a*) + *b* * *D*	Misconvergence								
66	*ln*(*B*) ∼ *ln*(*a*) + *b* * *D*^2^ * *H*									
67	*B*∼(*WD*/*a*)*exp(b*ln(*D*)+c*(*ln*(*D*))^2^+d*(*ln*(*D*)^3^)+*e*)									
68	*B*∼(*WD*/*a*)*exp(b*ln(*D*)+*c*)									
69	*B*∼*exp*(*a*+b*ln(*D*)+c*(*ln*(*D*))^2^+d*ln(*H*)+e*ln(*G*))									
70	*B* ∼ *a* * *H*^*b*^ * (*D* + 1)^(*c*+d*ln(*D*))^									
71	*B* ∼ *a* * *D*^2^ + (*D*^2^ − *b*) * *c*									
72	*ln*(*B*) ∼ *a* + *b* * *ln*(*D*) + *c* * *ln*(*D*^2^) + *d* * *ln*(*H*)									
73	*B* ∼ *exp*(*a*+*b* * *ln*(*D*)) + *exp*(*c* + *d* * *ln*(*D*))									
74	*B* ∼ *a* + (*b* * (1/*D*^2^) + *c* * (1/*D*^2^)) * *D*^2^									

a, b, c, d, e, f are the model parameters. RMSE, MAD and R^2^are model evaluation indexes.V is stem volume (m^3^). B is the whole tree biomass (kg). D is the diameter at breast height (cm). H is the tree total height (m). G is a basal area (m^2^). BCEF is the biomass wood density conversion factor, (i.e., the ratio of aboveground biomass over stem volume (kg*m^-3^)). BEF is the biomass expansion factor (i.e., the ratio of aboveground biomass over trunk biomass and is dimensionless). *BCEF* = *BEF* * *WD*. WD is wood density(the dry weight per unit volume of wood (kg*m^-3^)). Ln denotes the natural logarithm.

### 3.2Modified total tree biomass model

**Fig 3:**
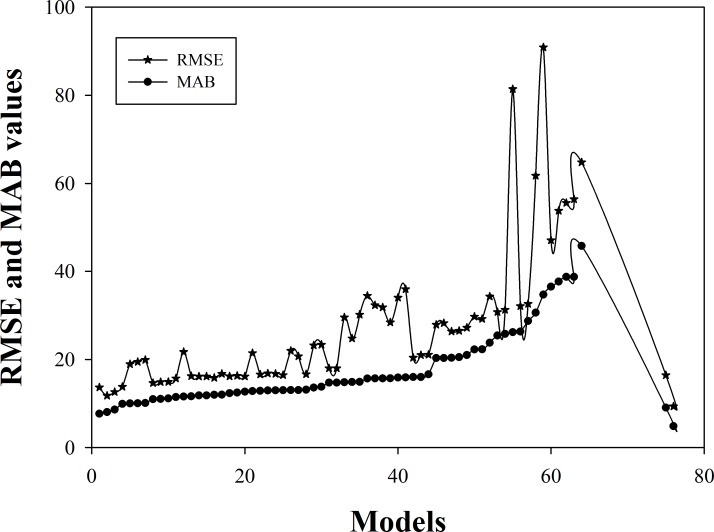
MAB and RMSE values of different biomass estimation models.

1. Based on the above analysis, the natural logarithms can be incorporated into the mathematical model’s structure.
2. The parameters of the model are summarized by the 3 indices of D, H and WD [13,21].
3. The size of the trees can be described by the forest measurements D and H, and D and H are comprehensive statistics for the volume (TV)[27].
4. According to (1), (2), and (3), an improved expression can be written as follows:
  ln(TB)=a+b*ln(TV)+c*ln(WD) (9)
  After an analysis of fit, a = 3.5743, b = 0.8887, c = 0.4106, MAB = 9.051, RMSE = 16.424, R^2^ = 0.975.
  A comprehensive comparison of model No.5 (with 3 variables) and Eq 9, under the same conditions as the 3-variable model, indicates that the evaluation indicators RMSE and R^2^ are similar, but the mean absolute bias of Eq 9 is less than that of model No.5 (0.956). Compared to the other models, the stand variable in Eq 9 is easy to measure and better explains the biomass, which has an apparent relationship between tree volume and wood density. After this step, the accuracy was less than that of model No.1, so Eq 9 is not the best biomass model. Therefore, Eq 9 must be modified.
5. In analysis (4), Eq 9 performed very well using an expression for V, as shown in Fang’s study[31], which indicates that a particular type of biomass is closely associated with the timber volume ratio (BEF)[1]. The Equation *BCEF* = *BEF* * *WD* was incorporated into Eq 9 in the accumulation variable BECF[18], thus introducing the parameters contained in BECF. Eq 9 can now be written as follows:
  ln(TB)=a+b*ln(TV)+c*ln(WD)+d*ln(BECF) (10)
  which was designated as Eq 10.
  A fit analysis of Eq 10 indicated that a = -0.0703, b = 0.9780, c = 0.9365, d = 0.0213, MAB = 3.7799, RMSE = 5.8135, and R^2^ = 0.997.
6. A comparative analysis of model No.1 and Eq 10 showed that, after incorporating the biomass conversion factor BECF, the MAB dropped to 4.8483, which was less than that of model No.1 by 2.8267.The RMSE decreased to 7.09, less than that of model No.1 by 6.566, and the R^2^ increased by 0.017. Models that included the variable BECF showed a significantly increased accuracy.
7. The above analysis indicates that Eq 10 is the optimal tree biomass equation for Chinese Fir(Fig 3), as follows:
  $$\begin{array}{l} ln\left(TB\right)=-0.0703+0.9780\;*\;ln\left(TV\right)+\\ 0.0213\;*\;ln\left(WD\right)+1.0166\;*\;ln\left(BECF\right) \end{array}$$ (11)

### 3.3 Development of a stand biomass model

The wood density and conversion coefficient combined with a different volume size can be used to estimate the biomass of a species. The definition of a forest stand for a tree species indicates that the WD and BECF are constants[14]. Therefore, the unit stand biomass equation (bi) is as follows:

$$bi=Exp\left(\begin{array}{l} -0.0703+0.9780\;*\;ln\left(TV\right)+\\ 0.0213*ln\left(WD\right)+1.0166*ln\left(BECF\right) \end{array}\right)$$ (12)

In this study, bi is defined as the stand biomass coefficient[12]. The stand biomass equation can be written as follows:

SB=bi*SV/TV (13)

where SV is the stand volume (m^3^),SB is the stand biomass (kg), and TV is the sample tree volume (m^3^).

The parameter n is defined as *n* = *SV* / *TV*, which can be used to obtain the following:

SB=bi*n (14)

**Fig 4:**
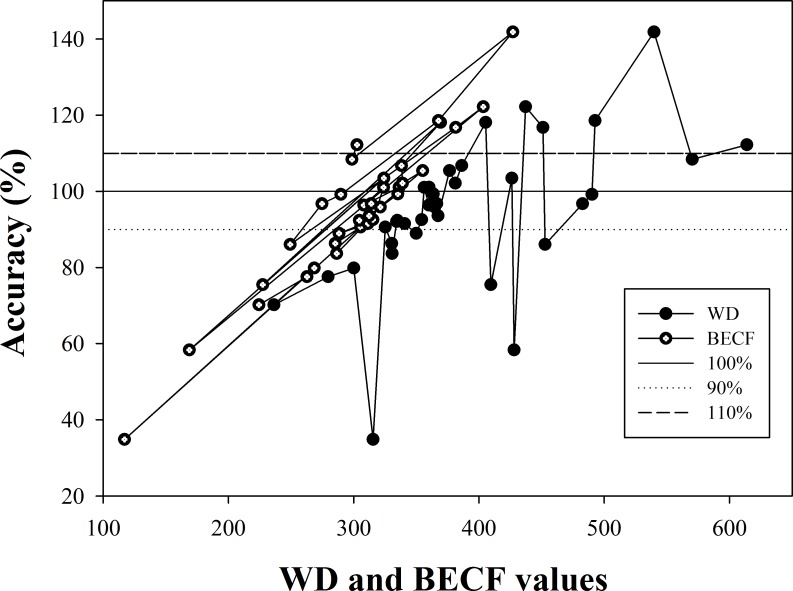
Changes in the model accuracy with parameters WD and BECF.

In this new equation, the amount of parameter is less than in model No.1, making it highly significant in forestry(Fig 4).

The precision of the model is stable for 35 types of trees, and the highest accuracy was obtained when BECF ranged from 300 to 350.For a value of WD ranging from 350 to 400, the accuracy was as high as 90% (Fig 4). A BECF less than 363.49 led to an estimated value less than the experimental value or greater than the measured value [32]. These parameters are easy to obtain, so this method is highly feasible.

### 3.4 Total tree biomass model validation

Base on the jackknife method, the MAB = 3.7029, RMSE = 5.8067, R^2^ = 0.9945,AIC = -86.6, and BIC = -79.3. The values of evaluation indices MAB, RMSE, R^2^, AIC, BIC of in (Eq 15) are similar to (Eq 11). The total tree biomass equation is:

$$\begin{array}{l} ln\left(TB\right)=0.0102+0.9822*ln\left(TV\right)-\\ 0.01153*ln\left(WD\right)+1.03617*ln\left(BECF\right) \end{array}$$ (15)

## Discussion

In this study, we constructed a stand biomass estimation model for Chinese Fir from models in previous studies. Compared with the best previous biomass model, the precision of our model is higher, and the absolute bias in the mean is nearly 3-foldlower for Chinese Fir (Fig 3).

The new model includes the stem volume, wood density, and biomass wood density conversion coefficient BECF. The variables D and H are included in the stock volume estimation variable V, so the model explains the key elements that affect the biomass. The forest tree total biomass model also contains the aboveground and belowground biomass. With the total tree biomass as the dependent variable, the model estimates all biomass components of a tree, which gives the model the advantage of compatibility. This model more accurately estimates the biomass in comparison to a model that uses one part of a single tree[33].For Chinese Fir, a biomass estimation model to estimate forest biomass must use the biomass of the entire tree or the total diameter at breast height, the tree height and the basal area. However, this type of estimation is difficult as well as has too great a variance in the estimates. It is therefore more accurate to calculate the tree and stand biomass using the volume of a single tree.

A different analysis strategy for a different age structure coefficient of Chinese Fir plantations provides the stand biomass bi, and this series of parameters can be used to estimate the forest stand biomass for stands of various sizes. The dynamic stand volume can be combined with the site index and age estimates of growth, and the formula for the stand volume (SV) forecast can be used to perfect the forest biomass estimation model using easily obtained stand measurement variables[11].

**Fig 5:**
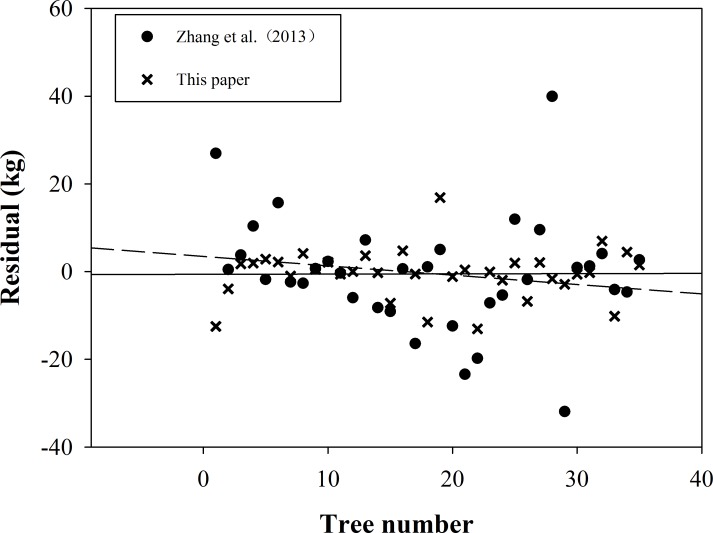
An accuracy comparison between Zhang et al. and this study.

Fang applied the biomass conversion factor (BEF) for large-scale biomass estimates, but we used the biomass wood density and conversion factor BCEF (*BCEF* = *BEF* * *WD*) to estimate the stand biomass. In different species, the accuracy of model in this study is higher than models No.5, No.6, No.7 and No.41 (Table 2) that have variable wood densities. We also showed that inter-specific variation of the wood density is the primary driver of biomass differences between species of similar sizes [13,15]. For the same species, compared to the biomass model built by Zhang et al. (2013) for Chinese Fir, the new model established in this paper that uses these new estimation variables for small-scale stands is more precise (Fig 5, Table 3).

**Table 3:** The evaluation indices used by Zhang et al. and in this study.

No	Model	AIC	BIC	MAB	RMSE	R^2^
This study	*ln*(*B*) = −0.0703+0.9780 * *ln*(*TV*)+0.9365*ln(*WD*) + 0.0213*ln(*BECF*)	-90.3	-82.8	3.7799	5.8135	0.997
Zhang et al. [34]	*B* = 0.0618 * (*D*^2^ * *H*)^0.8532^	269.7	274.2	8.6784	13.1613	0.987

In this study, we proposed a new forest biomass model, *B* = *bi* * *n*, where bi is the first variable proposed for use with different tree species. The use of the tree volume taken from forest management data to calculate the bi of different species is very important for estimates at different scales [35]. The relationship between the new parameter bi and other stand indicators still requires further detailed study [36].

## Conclusions

A combination of the stem volume(TV), the diameter at breast height (D), the tree total height (H), the biomass wood density conversion factor (BCEF), the wood density (WD), and the natural logarithm produced the most accurate model of tree biomass: *ln*(*TB*) = −0.0703 + 0.9780 * *ln*(*TV*) + 0.0213 * *ln*(*WD*) + 1.0166 * *ln*(*BECF*).

We provided the first available model for stand biomass. For different species, it is necessary to first calculate the stand biomass coefficient bi, and then the stand biomass can be easily estimated using the formula *SB* = *bi* * *n*. The model has high precision, and the parameter is less than that of model No.1, which indicates that this model is a highly significant model for forestry and tree biology. The model more precisely estimated the stand biomass when bi, a BECF from 300 to 350, and a WD from 350 to 400 trees were used. These parameters are easy to obtain, and the model is easy to use. The model is very useful for evaluating the ecological benefit of forest planning and can be useful for carbon stock and sequestration assessments in fast-growing plantations.

## Supporting Information

S1 File The basic data of biomass. This is the S1 File legend.
(TXT)

S2 File The R code for the data analysis. This is the S2 File legend.
(DOCX)
