# Supplementary material for: Models for Predicting the Biomass of Cunninghamialanceolata Trees and Stands in Southeastern China
Source: PLoS One. 2017 Jan 17;12(1):e0169747. doi: 10.1371/journal.pone.0169747 (PMC5241017; doi:10.1371/journal.pone.0169747)
Supplement: S2 File — This is the S2 File legend. (DOCX) [file pone.0169747.s002.docx]

R code

x=read.table("SG.txt",header=T);attach(x);library(nlme)

f.nls=nls(log(B)~a +b*log(V)+c*log(WD)+d*log(BECF),data=x,start=list(a=0.3766,b=0.9685,c=0.9365,d=0.1538))

summary(f.nls)

n=32

m=4

p=predict(f.nls)

write.table((p),file="M7F.txt")

U=exp(p)

cc=U-B

jdcc=abs(cc)

MAB=sum(jdcc)/n

MAB

RMSE=sqrt(sum((cc)^2)/(n-1))

RMSE

MEF=1-(((n-1)*sum((cc)^2))/((n-m)*sum((B -mean(B))^2)))

MEF

B.mean=mean(B)

R2=1-((sum((cc)^2))/(sum((B - B.mean)^2)))

R2

AIC(f.nls)

BIC(f.nls)

logLik(f.nls)
